# The gut microbiota-derived metabolite trimethylamine *N*-oxide is elevated in Alzheimer’s disease

**DOI:** 10.1186/s13195-018-0451-2

**Published:** 2018-12-22

**Authors:** Nicholas M. Vogt, Kymberleigh A. Romano, Burcu F. Darst, Corinne D. Engelman, Sterling C. Johnson, Cynthia M. Carlsson, Sanjay Asthana, Kaj Blennow, Henrik Zetterberg, Barbara B. Bendlin, Federico E. Rey

**Affiliations:** 1 Wisconsin Alzheimer’s Disease Research Center, University of Wisconsin School of Medicine and Public Health, Madison, WI USA; 2 Department of Bacteriology, University of Wisconsin-Madison, Madison, WI USA; 3 Department of Population Health Sciences, University of Wisconsin School of Medicine and Public Health, Madison, WI USA; 4 Geriatric Research Education and Clinical Center, William S. Middleton Memorial Veterans Hospital, Madison, WI USA; 5 Institute of Neuroscience and Physiology, Department of Psychiatry and Neurochemistry, The Sahlgrenska Academy at the University of Gothenburg, Mölndal, Sweden; 6 Clinical Neurochemistry Laboratory, Sahlgrenska University Hospital, Mölndal, Sweden; 7 Department of Neurodegenerative Disease, University College London Institute of Neurology, Queen Square, London, UK; 8 UK Dementia Research Institute at University College London, London, UK; 9 Present Address: Department of Cellular and Molecular Medicine, Cleveland Clinic, Cleveland, OH USA

**Keywords:** Alzheimer’s disease, Cerebrospinal fluid, Biomarkers, Trimethylamine *N*-oxide, Microbiota, Gut bacteria, Amyloid, Tau, Neurofilament light

## Abstract

**Background:**

Trimethylamine *N*-oxide (TMAO), a small molecule produced by the metaorganismal metabolism of dietary choline, has been implicated in human disease pathogenesis, including known risk factors for Alzheimer’s disease (AD), such as metabolic, cardiovascular, and cerebrovascular disease.

**Methods:**

In this study, we tested whether TMAO is linked to AD by examining TMAO levels in cerebrospinal fluid (CSF) collected from a large sample (*n* = 410) of individuals with Alzheimer’s clinical syndrome (*n* = 40), individuals with mild cognitive impairment (MCI) (*n* = 35), and cognitively-unimpaired individuals (*n* = 335). Linear regression analyses were used to determine differences in CSF TMAO between groups (controlling for age, sex, and *APOE* ε4 genotype), as well as to determine relationships between CSF TMAO and CSF biomarkers of AD (phosphorylated tau and beta-amyloid) and neuronal degeneration (total tau, neurogranin, and neurofilament light chain protein).

**Results:**

CSF TMAO is higher in individuals with MCI and AD dementia compared to cognitively-unimpaired individuals, and elevated CSF TMAO is associated with biomarkers of AD pathology (phosphorylated tau and phosphorylated tau/Aβ_42_) and neuronal degeneration (total tau and neurofilament light chain protein).

**Conclusions:**

These findings provide additional insight into gut microbial involvement in AD and add to the growing understanding of the gut–brain axis.

**Electronic supplementary material:**

The online version of this article (10.1186/s13195-018-0451-2) contains supplementary material, which is available to authorized users.

## Background

The human gut is home to trillions of microbes, including bacteria, eukaryotes, and viruses, that participate in a lifelong symbiotic relationship with their human hosts. Resident gut microbes perform essential functions for human health ranging from regulating nutrition and metabolism to influencing immune system development and function [1]. Gut microbes impact human health and disease at least in part by metabolizing dietary and host-derived substrates, and generating biologically active compounds including signaling compounds (e.g., agonists of G-protein coupled receptors), biological precursors, and toxins [2–4]. The microbial-derived metabolite trimethylamine *N*-oxide (TMAO) has been implicated in metabolic [5], cardiovascular [6, 7], and cerebrovascular [8] disease. The production of TMAO occurs via a two-step process. First, gut microbes enzymatically generate trimethylamine (TMA) from dietary constituents such as choline or l-carnitine [9]. TMA then enters the circulation and is oxidized to TMAO in the liver by flavin-containing monooxygenase 1 and 3 (FMO1 and FMO3) [6]. A recent study [10] demonstrated that TMAO is measurable in cerebrospinal fluid (CSF), suggesting that this microbial-derived metabolite reaches the central nervous system (CNS), and may therefore be relevant to neurological function or disorders. Indeed, mice treated with dietary TMAO show increased brain aging and cognitive impairment, likely due to increased oxidative stress, mitochondrial dysfunction, and inhibition of mammalian target of rapamycin (mTOR) signaling in the brain [11].

Alzheimer’s disease (AD) pathology is characterized by extracellular beta-amyloid (Aβ) plaques and intracellular neurofibrillary tangles composed of hyperphosphorylated tau protein [12]. The underlying etiology of AD is highly complex and multifactorial. A variety of genetic and environmental factors have been implicated in AD etiopathogenesis, including contributions from gut microbiota [13–15]. While it has been hypothesized that TMAO could be associated with AD pathology [16], this relationship has not yet been fully investigated in humans with Alzheimer’s clinical syndrome (AD dementia) [17]. In this study, we examined levels of TMAO in a large sample of CSF collected from individuals with AD dementia, individuals with mild cognitive impairment (MCI), and cognitively-unimpaired individuals. We also investigated the relationships between CSF TMAO, AD biomarkers (Aβ and phosphorylated tau), and biomarkers of neuronal and synaptic degeneration (total tau, neurofilament light chain protein, and neurogranin). We found that CSF TMAO levels are elevated in individuals with AD dementia, and that elevated CSF TMAO is associated with elevated AD pathology and neuronal degeneration as measured in CSF.

## Methods

### Participants

We identified 414 individuals in the Wisconsin Alzheimer’s Disease Research Center (ADRC) clinical core (*n* = 277) and the Wisconsin Registry for Alzheimer’s Prevention (WRAP) study (*n* = 137) who had undergone lumbar puncture with CSF collection, as well as TMAO and biomarker quantification. The ADRC clinical core study consists of participants who fall along the clinical continuum of cognitive function, including AD dementia, MCI, and cognitively-unimpaired controls. The WRAP study is a large (> 1500 subjects), ongoing (> 15 years), prospective longitudinal investigation of the genetic, biological, and lifestyle factors that contribute to the development of AD dementia and cognitive decline [18]. Individuals in the WRAP study were recruited as cognitively-unimpaired, asymptomatic middle-aged adults and undergo biannual comprehensive medical and cognitive evaluation. Because both the WRAP study and the ADRC clinical core are enriched for risk of late-onset AD (~ 70% of WRAP subjects have a parental family history of AD, and ~ 50% of participants 45–65 years old in the ADRC study have a parental history of AD), the *APOE* ε4 genotype is more prevalent. General exclusion criteria for the ADRC and WRAP studies include any significant neurologic disease (other than AD dementia), history of alcohol/substance dependence, major psychiatric disorders (including untreated major depression), or other significant medical illness. *APOE* ε4 genotyping procedures have been described previously [19], and participants were categorized as noncarriers (zero ε4 alleles) or *APOE* ε4 carriers (one or two ε4 alleles). The University of Wisconsin Health Science Institutional Review Board approved all study procedures, and all experiments were performed in accordance with relevant guidelines and regulations. All participants provided written informed consent to be involved in this study.

### Diagnostic classification

Participants underwent a comprehensive neuropsychological battery to determine their cognitive status. Participants with MCI and AD dementia were diagnosed using available clinical and cognitive information in accordance with the updated 2011 National Institute on Aging–Alzheimer’s Association workgroup diagnostic criteria [20, 21]. All participants in the ADRC clinical core are discussed at a consensus review committee consisting of physicians, neuropsychologists, and nurse practitioners. Biomarker data are not used in determining clinical diagnosis. Participants in the WRAP study are reviewed selectively when flagged after cognitive abnormalities are detected by algorithm on neuropsychological tests, at which point cases are discussed at a consensus review committee meeting [18]. Of the 414 identified participants, four individuals with a diagnosis of nonneurodegenerative cognitive impairment at the time of CSF collection were excluded from the present analyses, resulting in a total of 410 participants: *n* = 335 cognitively-unimpaired participants (Control group), *n* = 35 MCI (MCI group), and *n* = 40 AD dementia (AD group).

### Lumbar puncture and CSF collection

Lumbar puncture and CSF collection procedures have been described previously [22]. Briefly, CSF was collected via lumbar puncture in the morning after a 12-h fast with a Sprotte 25 or 24-gauge spinal needle at the L3/4 or L4/5 interspace using gentle extraction into propylene syringes. CSF (~ 22 ml) was then combined, gently mixed, and centrifuged at 2000 × *g* for 10 min. Supernatants were frozen in 0.5 ml aliquots in polypropylene tubes and stored at − 80 °C.

### CSF biomarker quantification

CSF AD biomarkers included the Aβ_42_/Aβ_40_ ratio, phosphorylated tau (p-tau), and the p-tau/Aβ_42_ ratio. CSF Aβ is an indicator of amyloid burden, with greater amyloid deposition in the brain being reflected by lower levels in the CSF. The Aβ_42_/Aβ_40_ ratio (which normalizes CSF Aβ_42_ for the total amount of Aβ peptides that are present in CSF) was used given that it shows better correspondence with brain amyloid deposition as well as superior diagnostic performance compared to CSF Aβ_42_ alone [23]. p-tau is a marker of tau phosphorylation believed to be associated with neurofibrillary tangle pathology, with higher levels reflecting a more intense tau phosphorylation process; the ratio of p-tau/Aβ_42_ incorporates both facets of pathology, with higher values indicating greater AD pathology [24]. For the Aβ_42_/Aβ_40_ ratio, CSF Aβ_42_ and CSF Aβ_40_ were quantified separately by electrochemiluminescence (ECL) using an Aβ triplex assay (MSD Human Aβ peptide Ultra-Sensitive Kit; Meso Scale Discovery, Gaithersburg, MD, USA). For p-tau and the p-tau/Aβ_42_ ratio, CSF p-tau and Aβ_42_ were quantified using commercially available sandwich ELISAs (INNOTEST β-amyloid1–42 and Phospho-Tau[181 P], respectively; Fujirebio Europe, Ghent, Belgium).

CSF biomarkers of neuronal degeneration included total tau (t-tau), neurofilament light chain protein (NFL, a marker of axonal degeneration), and neurogranin (a marker of synaptic degeneration). CSF t-tau and NFL were quantified using commercially available sandwich ELISAs: t-tau, INNOTEST hTau Ag (Fujirebio Europe); and NFL, NF-Light ELISA kit (Uman Diagnostics AB, Umeå, Sweden). CSF neurogranin was quantified using a sandwich ELISA as described previously [25]. All CSF assays were performed in two batches (*n* = 192 samples in batch 1, *n* = 218 samples in batch 2), and all statistical analyses accounted for batch variation (see Statistical analysis).

### CSF TMAO quantification

CSF TMAO was quantified via an untargeted plasma metabolomics analysis performed by Metabolon, Inc. (Durham, NC, USA) using ultrahigh performance liquid chromatography tandem mass spectrometry (UHPLC-MS) as described previously [26] (details presented in Additional file 1: Methods). All samples were sent to Metabolon in one shipment. Raw data were extracted, peak identified, and QC processed using Metabolon’s hardware and software. TMAO levels were expressed as scaled intensity units (SIU) using the QC-processed mass-to-charge ratio (*m/z*) area-under-the-curve values for TMAO and scaled to a median value of 1.

### Statistical analysis

Our analysis approach first examined differences in CSF TMAO levels between clinical diagnostic groups, and then extended these analyses in order to characterize the biological relationships between CSF TMAO and biomarkers of both AD pathology and neurodegeneration. To determine CSF TMAO differences between groups, a multiple linear regression model was conducted in R (v3.5.0) to test the effect of age, sex, *APOE* ε4 genotype, and clinical diagnosis (Control, MCI, AD dementia) on CSF TMAO levels. CSF TMAO was natural log transformed to account for a nonnormal distribution. Secondarily, linear regression models were used to determine the relationship between CSF TMAO and CSF biomarkers (Aβ_42_/Aβ_40_, p-tau, p-tau/Aβ_42_ ratio, t-tau, NFL, and neurogranin). Separate models were run for each CSF biomarker, and each model included covariates of age, sex, and the nuisance covariate of CSF analysis batch (to account for batch variation). Given that TMAO has been implicated in cardiovascular disease, and that vascular disease risk factors are associated with AD and neurodegeneration, the same linear regression models were run for each CSF biomarker with the addition of peripheral vascular disease measures as covariates (BMI, blood pressure, total cholesterol, HDL cholesterol, and fasting glucose). Nonnormally distributed variables were natural log transformed.

## Results

### Participant characteristics

Participant characteristics are reported in Table 1. The Control group tended to be younger and had a higher proportion of females compared to the MCI and AD dementia groups. As expected, the *APOE* ε4 genotype was more prevalent in the MCI and AD dementia groups. There were no differences between groups with respect to cardiovascular disease risk factors including BMI, blood pressure, total cholesterol, HDL cholesterol, and fasting glucose.

**Table 1:** Participant characteristics

Sample characteristic	All	Control	MCI	AD dementia
*N*	410	335	35	40
Age (years)	63.8 ± 9.0	61.9 ± 7.9	73.2 ± 8.5	71.9 ± 8.6
Sex (% female)	63 (258/410)	69 (231/335)	31 (11/35)	40 (16/40)
*APOE* ε4 genotype, % positive (*n*)	43 (178/410)	39 (130/335)	54 (19/35)	73 (29/40)
AD parental history, % positive (*n*)	58 (236/410)	64 (214/335)	31 (11/35)	28 (11/40)
Ethnicity, % Caucasian (*n*)	97 (396/410)	96 (321/335)	100 (35/35)	100 (40/40)
Education (years)	15.9 ± 2.6	16.1 ± 2.5	16.3 ± 2.7	14.7 ± 2.8
Body mass index (BMI)	28.3 ± 5.5	28.5 ± 5.7	28.2 ± 4.4	26.6 ± 3.9
Systolic blood pressure (mmHg)	128 ± 17	125 ± 16	130 ± 18	134 ± 16
Diastolic blood pressure (mmHg)	74 ± 9	75 ± 9	76 ± 10	75 ± 9
Total cholesterol (mg/dl)	195 ± 37	199 ± 46	184 ± 44	190 ± 37
HDL cholesterol (mg/dl)	62 ± 18	64 ± 39	57 ± 19	56 ± 14
Fasting glucose (mg/dl)	97 ± 20	99 ± 51	99 ± 15	100 ± 18
CSF data				
TMAO (SIU)	1.5 ± 1.7	1.3 ± 1.5	2.1 ± 1.4	2.8 ± 2.9
p-tau (pg/ml)	49.3 ± 22.0	45.1 ± 16.4	61.3 ± 36.2	74.7 ± 26.9
p-tau/Aβ_42_	0.09 ± 0.07	0.07 ± 0.04	0.14 ± 0.11	0.21 + 0.09
Aβ_42_/Aβ_40_	0.086 ± 0.024	0.090 ± 0.022	0.076 ± 0.028	0.062 ± 0.022
t-tau (pg/ml)	370 ± 228	310 ± 141	552 ± 338	722 ± 300
NFL (pg/ml)	841 ± 655	705 ± 489	1247 ± 863	1628 ± 935
Neurogranin (pg/ml)	352 ± 208	328 ± 182	430 ± 308	488 ± 244

All data presented as mean ± standard deviation unless otherwise indicated

*MCI* mild cognitive impairment, *AD* Alzheimer’s disease, *APOE* ε4 apolipoprotein E epsilon 4 allele, *HDL* high-density lipoprotein, *CSF* cerebrospinal fluid, *TMAO* trimethylamine *N*-oxide, *SIU* scaled intensity units, *p-tau* phosphorylated tau, *Aβ* beta-amyloid, *t-tau* total tau, *NFL* neurofilament light chain protein

### CSF TMAO is elevated in individuals with MCI and AD dementia

**Fig. 1:**
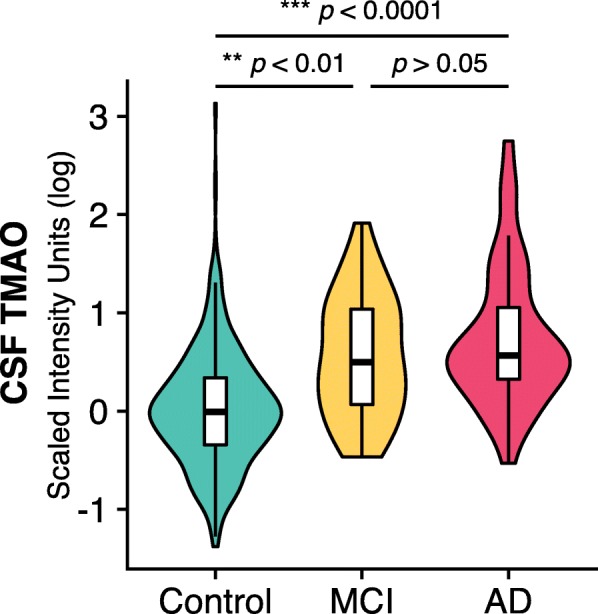
CSF TMAO levels are elevated in individuals with AD dementia and MCI compared to cognitively-unimpaired individuals, after controlling for age, sex, and *APOE* ε4 genotype. Data presented as violin plots (displaying scaled distribution of data for each group) with inset Tukey boxplots showing median, interquartile range (IQR), and 1.5 × IQR. AD Alzheimer’s disease, CSF cerebrospinal fluid, MCI mild cognitive impairment, TMAO trimethylamine *N*-oxide

CSF TMAO levels were elevated in individuals with AD dementia (*β* = 0.50, *p* < 0.0001) and MCI (*β* = 0.29, *p* < 0.05) compared to cognitively-unimpaired individuals (Fig. 1; Table 2), controlling for age, sex, and *APOE* ε4 genotype. Older age was associated with higher CSF TMAO (*β* = 0.02, *p* < 0.0001), but there were no main effects of sex or *APOE* ε4 genotype, and CSF TMAO levels did not differ between the MCI and AD groups.

**Table 2:** Summary of multiple linear regression of age, sex, *APOE* ε4 genotype, and diagnosis on CSF TMAO level

Variable	*β* (standard deviation)	*t*	*p* value
Age	0.022 (0.004)	6.1	5.6 × 10^−8^***
Sex	−0.02 (0.06)	−0.03	0.75
*APOE* ε4 genotype	−0.02 (0.06)	−0.3	0.77
Diagnosis			
Control vs MCI	0.29 (0.11)	2.6	0.01*
Control vs AD	0.50 (0.11)	4.7	4.1 × 10^− 6^***
MCI vs AD	0.21 (0.13)	1.5	0.12

*APOE* ε4 apolipoprotein E epsilon 4 allele, *MCI* mild cognitive impairment, *AD* Alzheimer’s disease

Overall model statistics: *F*_5,404_ = 23.1, adjusted *R*^2^ = 0.21, *p* < 2.2 × 10^− 16^

*p < 0.05

***p < 0.001

### CSF TMAO is associated with CSF biomarkers of AD and neuronal degeneration

**Fig. 2:**
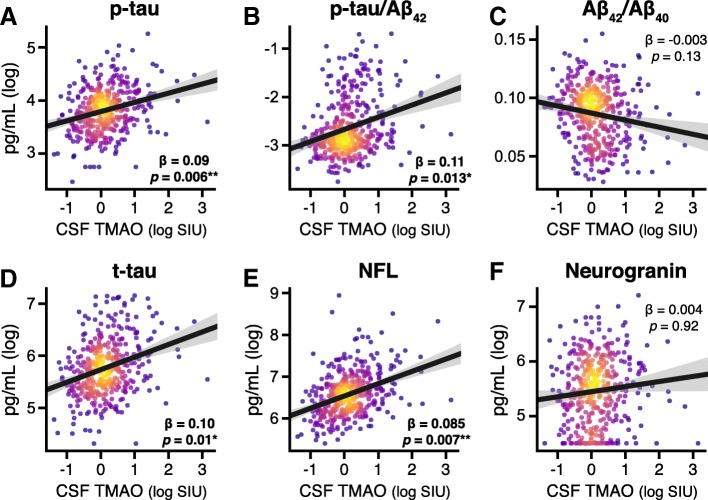
Relationship between CSF TMAO and CSF AD biomarkers (**a**–**c**) and biomarkers of neuronal degeneration (**d**–**f**). CSF TMAO is significantly positively correlated with phosphorylated tau (p-tau), p-tau/Aβ_42_, total tau (t-tau), and neurofilament light chain protein (NFL), after controlling for age and sex. Scatterplots show individual data points (*n* = 410) colored by 2D kernel density estimation. Hotter colors represent higher density; black line represents best linear fit between variables; shading represents 95% confidence interval of fit. CSF TMAO expressed as natural log-transformed scaled intensity units (SIU). Aβ, beta-amyloid CSF cerebrospinal fluid, TMAO trimethylamine *N*-oxide

With respect to CSF AD biomarkers, there was a significant positive relationship between CSF TMAO and p-tau (*β* = 0.09, *p* = 0.006; Fig. 2a) and p-tau/Aβ_42_ (*β* = 0.11, *p* = 0.013; Fig. 2b). No significant relationship between CSF TMAO and Aβ_42_/Aβ_40_ (*β* = − 0.003, *p* = 0.13; Fig. 2c) was observed. Additionally, CSF TMAO was positively associated with both CSF t-tau (*β* = 0.10, *p* = 0.01; Fig. 2d) and CSF NFL (*β* = 0.085, *p* = 0.007; Fig. 2e), but there was no relationship between CSF TMAO and CSF neurogranin (*β* = 0.004, *p* = 0.92; Fig. 2f). Additional file 1: Figure S1 shows the relationships between CSF TMAO and biomarkers colored by diagnostic group. Including peripheral cardiovascular disease risk factors as covariates did not change these associations (see Additional file 1: Table S1).

## Discussion

Understanding the contributions of the gut microbiota to neurological function and disease is an expanding area of research, particularly with respect to neurodegenerative disorders. A recent study [16], which used publicly available databases and a data-driven hypothesis-free computational approach to address the links between gut microbiota and AD, proposed that the gut microbial-derived metabolite TMAO is highly associated with AD. In the present study, we provide biochemical evidence revealing that CSF TMAO is higher in individuals with MCI and AD dementia, and elevated CSF TMAO is associated with both increased AD pathology (as measured by CSF biomarkers) as well as markers of neuronal degeneration.

Specifically, we found that CSF TMAO was associated with CSF p-tau as well as p-tau/Aβ_42_, but not Aβ_42_/Aβ_40_, potentially indicating that TMAO is more closely related to tau pathology than amyloid deposition alone. Additionally, we examined CSF biomarkers of neuronal degeneration, including t-tau, NFL, and neurogranin. CSF t-tau and NFL are thought to reflect axonal integrity [27] (with higher levels indicating greater axonal degeneration), while neurogranin is expressed in dendritic spines and reflects synaptic integrity [24]. We found that CSF TMAO was associated with increased CSF t-tau and NFL, but not neurogranin, suggesting that TMAO is related to axonal injury, but not dendritic degeneration. Taken together, our results suggest that while TMAO may not be a primary driver of amyloid production, it may impact vulnerable neurons and contribute to neurodegeneration.

As a metaorganismal metabolite, the production and accumulation of TMAO is dependent on both bacterial and host metabolism. The gene cluster required for bacterial enzymatic conversion of choline to TMA is distributed widely and discontinuously among gut bacterial taxa [9, 28, 29]. Thus, the presence of TMA-producing bacteria cannot be predicted from bacterial 16S rRNA gene sequencing studies. In the host, oxidation of TMA via FMO3 in the liver can also regulate TMAO levels [30]. Additionally, while both vegetarians and omnivores are able to convert choline to TMA [7, 31], long-term dietary habits can influence TMAO accumulation via changes in gut microbiota composition, which modulates TMA production potential.

TMAO is thought to contribute to disease pathogenesis through a variety of mechanisms including altering lipid and hormonal homeostasis, promoting platelet hyperreactivity [8], modulating cholesterol and sterol metabolism, decreasing reverse cholesterol transport [7], and inducing endothelial dysfunction through activation of the NLRP3 inflammasome [32]. In the brain, TMAO has been shown to induce neuronal senescence, increase oxidative stress, impair mitochondrial function, and inhibit mTOR signaling [11], all of which contribute to brain aging and cognitive impairment. Additionally, TMAO upregulates macrophage scavenger receptors and induces CD68 expression [7, 33], a cellular marker positively associated with dementia [34].

Vascular risk factors are increasingly recognized as important contributors to AD dementia [35], and cerebrovascular pathology commonly coexists with AD pathology at autopsy [36]. TMAO is causally linked with exacerbation of atherosclerosis in a genetically modified mouse model [6, 7], and the presence of intracranial atherosclerosis is an independent risk factor for dementia [37]. Thus, one potential mechanism by which TMAO may play a role in AD pathology is through the promotion of cerebrovascular disease. Of note, in the present study, cognitively-unimpaired, MCI, and AD individuals did not differ with respect to cardiovascular disease risk factors (BMI, blood pressure, cholesterol, and fasting glucose), suggesting that differences observed in TMAO between groups did not reflect underlying differences in cardiovascular disease status. Moreover, controlling for peripheral vascular disease risk factors did not change the associations between CSF TMAO and biomarkers of AD and neurodegeneration, suggesting that TMAO may have an impact independent of vascular effects. However, our study did not examine direct measures of central vascular disease, and future studies are needed to more fully examine the relationship between TMAO and cerebrovascular health.

TMAO is elevated in individuals with diabetes [38] and has been shown to promote insulin resistance in mice fed a high-fat diet [5]. Given that diabetes and insulin resistance are risk factors for developing AD [39, 40], elevated TMAO in the CNS may exacerbate central insulin resistance and AD pathogenesis. Finally, mitochondrial dysfunction and increased oxidative stress are ubiquitous features of AD pathology [41]; mice treated with dietary TMAO show increased brain aging with similar features [11], suggesting elevated TMAO may accelerate neurotoxicity and neurodegeneration in the context of AD pathology. However, additional work is needed to determine the potentially multifactorial pathways by which TMAO impacts the brain. Given that our results indicate that TMAO may be more relevant to neurodegenerative changes rather than initiation of Alzheimer’s-specific amyloid pathology, CSF TMAO levels should be investigated in other neurodegenerative disorders (e.g., Parkinson’s disease).

## Conclusions

In this study, we demonstrate that the gut microbiota-derived metabolite TMAO is elevated in the CSF of individuals with MCI and AD dementia, and that levels of CSF TMAO are associated with CSF biomarkers of AD pathology and neuronal degeneration. These results provide additional evidence for an association between TMAO and AD, and further inform the role of gut microbiota in AD. Longitudinal studies are needed to determine whether elevated TMAO during mid-life predicts subsequent development or exacerbation of AD pathology. In this scenario, pharmacological agents designed to inhibit gut microbial TMAO production may be useful in slowing AD pathology [42].

## Additional file

Additional file 1: **Methods.** Additional details from Metabolon for methodology of CSF TMAO quantification. **Figure S1.** Relationship between CSF TMAO and CSF AD biomarkers (A–C) and biomarkers of neuronal degeneration (D–F). **Table S1.** Summary of multiple linear regressions testing relationships between CSF TMAO and CSF biomarkers of AD pathology and neurodegeneration. (PDF 895 kb)

## Abbreviations

AD: Alzheimer’s disease
ADRC: Alzheimer’s Disease Research Center
*APOE* ε4: Apolipoprotein E epsilon 4 allele
Aβ: Beta-amyloid
CNS: Central nervous system
CSF: Cerebrospinal fluid
ECL: Electrochemiluminescence
ELISA: Enzyme-linked immunosorbent assay
FMO1: Flavin-containing monooxygenase 1
FMO3: Flavin-containing monooxygenase 3
MCI: Mild cognitive impairment
mTOR: Mammalian target of rapamycin
NFL: Neurofilament light chain protein
NLRP3: NACHT, LRR and PYD domains-containing protein 3
p-tau: Phosphorylated tau
SIU: Scaled intensity units
TMA: Trimethylamine
TMAO: Trimethylamine *N*-oxide
t-tau: Total tau
UHPLC-MS: Ultrahigh performance liquid chromatography tandem mass spectrometry
WRAP: Wisconsin Registry for Alzheimer’s Prevention
